# Trabecular bone score in active or former smokers with and without COPD

**DOI:** 10.1371/journal.pone.0209777

**Published:** 2019-02-01

**Authors:** Jessica González, Macarena Rodríguez-Fraile, Pilar Rivera, Patricia Restituto, Inmaculada Colina, María de los desamparados Calleja, Ana B. Alcaide, Aránzazu Campo, Juan Bertó, Luís M. Seijo, Teresa Pérez, Javier Zulueta, Nerea Varo, Juan P. de-Torres

**Affiliations:** 1 Pulmonary Department, Clínica Universidad de Navarra, Pamplona, Spain; 2 Nuclear Medicine Department and clinical densitometry certified, Clínica Universidad de Navarra, Pamplona, Spain; 3 Biochemical Analysis Department, Clínica Universidad de Navarra, Pamplona, Spain; 4 Department of Internal Medicine, Clínica Universidad de Navarra, Pamplona, Spain; 5 Department of Endocrinology & Nutrition, Clínica Universidad de Navarra, Pamplona, Spain; 6 Pulmonary Department, Clínica Universidad de Navarra, Madrid, Spain; Istituti Clinici Scientifici Maugeri, ITALY

## Abstract

**Background:**

Smoking is a recognized risk factor for osteoporosis. Trabecular bone score (TBS) is a novel texture parameter to evaluate bone microarchitecture. TBS and their main determinants are unknown in active and former smokers.

**Objective:**

To assess TBS in a population of active or former smokers with and without Chronic Obstructive Pulmonary Disease (COPD) and to determine its predictive factors.

**Methods:**

Active and former smokers from a pulmonary clinic were invited to participate. Clinical features were recorded and bone turnover markers (BTMs) measured. Lung function, low dose chest Computed Tomography scans (LDCT), dual energy absorptiometry (DXA) scans were performed and TBS measured. Logistic regression analysis explored the relationship between measured parameters and TBS.

**Results:**

One hundred and forty five patients were included in the analysis, 97 (67.8%) with COPD. TBS was lower in COPD patients (median 1.323; IQR: 0.13 vs 1.48; IQR: 0.16, p = 0.003). Regression analysis showed that a higher body mass index (BMI), younger age, less number of exacerbations and a higher forced expiratory volume-one second (FEV_1_%) was associated with better TBS (β = 0.005, 95% CI:0.000–0.011, p = 0.032; β = -0.003, 95% CI:-0.007(-)-0.000, p = 0.008; β = -0.019, 95% CI:-0.034(-)-0.004, p = 0.015; β = 0.001, 95% CI:0.000–0.002, p = 0.012 respectively). The same factors with similar results were found in COPD patients.

**Conclusions:**

A significant proportion of active and former smokers with and without COPD have an affected TBS. BMI, age, number of exacerbations and the degree of airway obstruction predicts TBS values in smokers with and without COPD. This important information should be considered when evaluating smokers at risk of osteoporosis.

## Introduction

Osteoporosis affects an estimated 200 million people, being a major cause of morbidity and mortality worldwide and resulting in approximately 9 million new fractures annually [1]. The agreed international definition of osteoporosis [2] highlights the notion that both low bone mineral density (BMD) and other bone abnormalities such as microarchitectural impairment contribute to skeletal fragility. However, the diagnosis of osteoporosis is based solely on the measurement of BMD by dual energy absorptiometry (DXA) scan [3]. Interestingly, an impaired bone microarchitecture, independent of BMD, is also associated with a greater risk of bone fracture [4]. Therefore, it is recognized that the evaluation of bone microarchitecture might enhance the accuracy of risk fracture assessment.

Lumbar spine trabecular bone score (TBS) was developed as an innovative grey-level texture parameter that can be applied to DXA acquisitions. TBS is calculated from experimental variograms of the 2D projection DXA images, quantifying variation in grey-level texture as a function of distance from one pixel to the others. It is not exactly a measurement of bone microarchitecture, but approximately represents the 3D bone characteristics: trabecular separation, trabecular number, and connectivity density [4,5]. Therefore, higher TBS corresponds to more homogeneously textured bone, representing strong and fracture resistant microarchitecture and vice versa. This novel score does not require further patient tests and is easily calculated in seconds by commercially available software applied prospectively or retrospectively to standard DXA images [6].

Smoking has long been identified as a risk factor for osteoporosis, with studies showing that older smokers have decreased bone mineral density measured by DXA scan and increased fracture risk compared to nonsmokers, particularly at the hip [7]. Moreover, the increase in fracture risk in this population is out of proportion to the alterations seen on bone density; this indicates, perhaps, a deficiency in bone quality [8].

To better understand the potential clinical utility of TBS in smokers, the main objective of this study is to describe TBS and its predictive factors in a population of active and former smokers with and without COPD.

## Materials and methods

### Participants

Study subjects were active and former smokers evaluated at the pulmonary clinic of Clínica Universidad de Navarra between August 2014 and March 2016. Invited patients were men and postmenopausal women, 50 years of age or older, with a smoking history of ≥10 pack-years. Previous diagnosis of osteoporosis, use of preventive osteoporosis treatment and ≥6 month between the DXA scan and the blood draw were the exclusion criteria. All subjects signed a consent form previously approved by the Institution’s ethics committee (Comite de Ética Clínica Universidad de Navarra, n°151/2014).

The initial visit included a complete medical history, physical examination, blood samples collection, pulmonary function tests (PFTs), 6 minute walking distance (6MWD), BMD measurement and a low-dose chest computed tomography (LDCT). A questionnaire was administered by a single investigator including age, race, body mass index (BMI), menopause information, personal and family fracture history, tobacco and alcohol intake history, medication use in the past and present (including oral and inhaled corticosteroids) and number of respiratory exacerbations. Respiratory exacerbations were defined as an acute worsening of respiratory symptoms (cough, sputum production, dyspnea, wheezing) which result in the use of additional therapy [9,10], as defined by global initiative for chronic obstructive lung disease (GOLD) [11].

**Fig 1:**
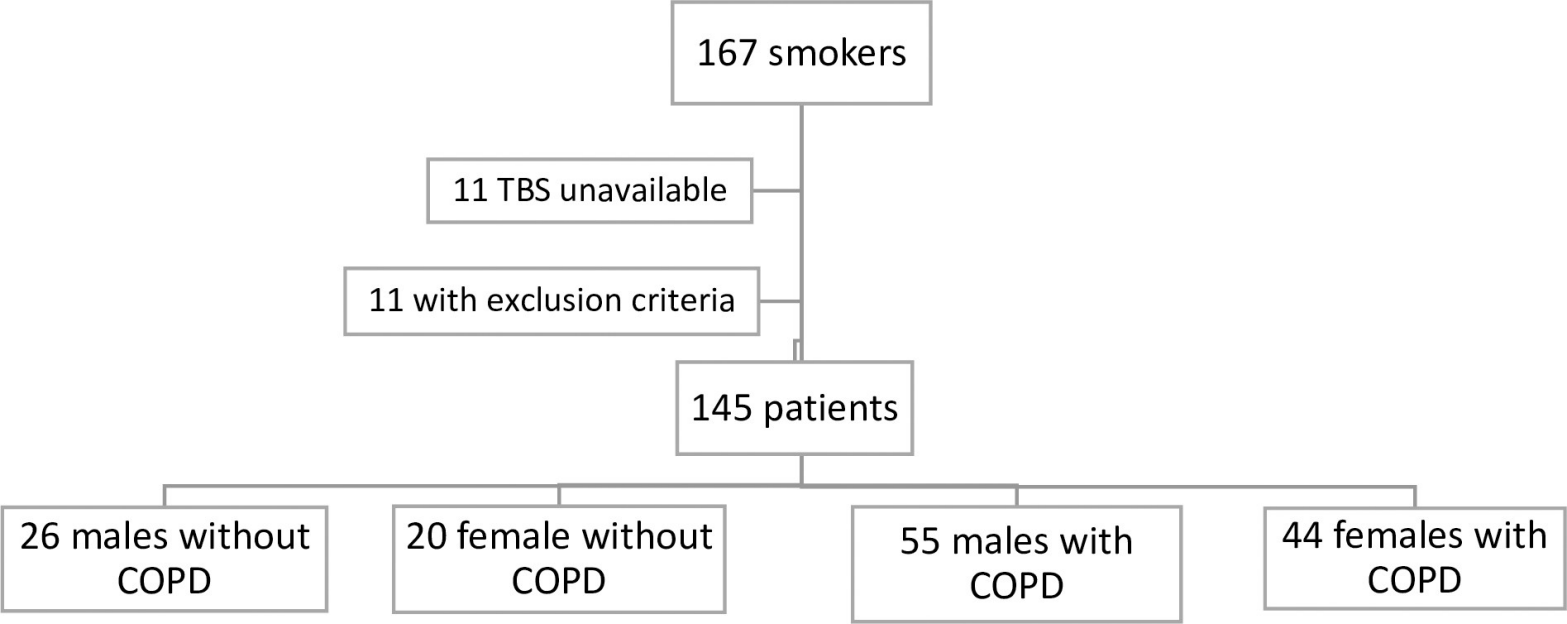
Study design. Flowchart showing the inclusion of the participants.

Fig 1 shows the Flowchart of the included individuals.

### Pulmonary function tests (PFTs)

PFTs (spirometry, lung volumes and diffusing capacity) were performed with a flow spirometer (Vmax22; SensorMedics, Yorba Linda, CA) according to the American Thoracic Society guidelines [12]. The European Community Lung Health Survey values were used as a reference [13]. Post bronchodilation measurements were determined 15´ after the inhalation of 400 μg of albuterol. The presence and severity of airflow limitation was determined using the GOLD definition and spirometric classification [11]. The latter is based on the predicted postbronchodilator forced expiratory volume-one second (FEV_1_) and is composed of four groups: 1 (FEV_1_≥80%), 2 (50–80%), 3 (30–50%), and 4 (<30%). The 6MWD was performed following the American Thoracic Society guidelines [14].

### Bone density by Dual X-ray absorptiometry

Bone mineral density of the lumbar spine (L1 to L4), femoral neck, total hip and in some cases non-dominant forearm (33%) were measured in every patient using a dual X-ray absorptiometry (DEXA) technique with a Lunar iDXA scan (General Electric Co). Results were expressed in g/cm^2^ and a T-score was calculated as the number of standard deviations above or below the young normal reference mean BMD. Diagnosis of osteoporosis was based on the World Health Organization (WHO) criteria [15].

### Trabecular bone score (TBS)

Lumbar spine TBS was obtained using the DXA images of each patient. TBS was calculated using TBS iNsight software (Version 2.2.0, Med-Imaps, Bordeaux, France) by one of the authors (MR) who was blinded to the clinical data of the participants. Since there is a lack of reference values for TBS in a Mediterranean population like the present one, we used the three categories recommended by the software provider (iNsight): 1) normal microarchitecture: TBS≥1.300; 2) partially deteriorated microarchitecture: TBS<1.300 and >1.200 and 3) degraded microarchitecture: TBS≤1.200.

### Low dose chest CT (LDCT)

LDCT examinations were obtained with the patient in supine position, in cranio-caudal direction and at end-inspiration.

Patients were studied with a 64 slice multidetector CT scanner (Somatom Sensation 64, Somatom Definition, Siemens Healthcare, Erlangen, Germany), also at a low-dose setting (120 kV tube voltage, 40 mAs tube current, 64x0.6 mm slice collimation, 0.5 s gantry rotation time, 1.4 pitch, 1 mm slice thickness, 1 mm reconstruction interval).

### Assessment of emphysema on LDCT

Emphysema was visually assessed in the chest CT by one reader (JG) for presence, type and severity, using a validated criteria established by the Fleischner Society [16]. Scoring procedures used a five-level semi quantitative scale based on criteria used in the National Emphysema Treatment Trial [17].

### Biochemistry

All the samples were drawn in all patients early in the morning of the visit day and in fasting status. Samples were collected into Vacutainer tubes, and aliquots were stored frozen at -80°C until analysis. Measurements of BTM were performed at the Laboratory of Biochemistry of the Clínica Universidad de Navarra. Details of the measurements have been previously described [18].

### Statistical analysis

Statistical analysis was performed using the Statistical Package for the Social Sciences version 20.0 (SPSS Inc). Normal distribution was assessed by the Shapiro-Wilks test. Quantitative data are represented as mean ± SD or median (interquartile range), depending on the data distribution; relative frequencies were used for qualitative data. Differences between study groups were evaluated by the Student’s *t* test, Mann-Whitney *U* test, and χ^2^ statistics accordingly. Simple bivariate Pearson correlation coefficients were examined between TBS and other continuous variables (age, BMI, lumbar spine BMD), showing a weak inverse correlation with age (-0.25), a moderate positive correlation with BMI (0.33) and a strong positive correlation with lumbar spine BMD (0.56). Uni and multivariable linear regression analysis with TBS as a dependent variable were performed to study the potential independent association of the studied parameters. The multivariable linear regression analysis included those variables that were statistically significant in the univariable analysis and those clinically soundable. Two multivariable analyses were performed, one in the entire population and another focused on COPD patients. All analysis was adjusted for age, sex, body mass index (BMI) and lumbar spine BMD, based on the correlation analysis.

## Results

The study population characteristics are shown in Table 1. This study sample included patients in their 6^th^ decade with a slight predominance of males (56%) and somewhat overweight (mean 27.2 Kg/m^2^). Two thirds of the patients met COPD criteria with a mild degree of airway limitation.

**Table 1:** Baseline clinical, bone remodeling markers and densitometry characteristics of the study population.

Characteristics	N = 145
**Demographic**	
Age years-old, Mean (SD)	63 (8)
Male, No(%)	81 (56)
BMI Kg/m^2^, Mean (SD)	27.2 (4.6)
**Clinical**	
Diabetes	20 (13.8)
Active smoker, No (%)	69 (48)
Pack-years of smoking, Median (IQR)	40.5 (28.5)
Emphysema, No (%)	82 (56.5)
Severity of emphysema by NETT (0–4), Median (IQR)	1(2)
Inhaled steroids, No (%)	37 (26)
COPD, No (%)	97 (67.8)
BODE index for COPD patients, Median (IQR)	0 (1)
**Post-Bronchodilator spirometry**	
FVC-L, Mean (SD)	3.66 (1.2)
FVC-%, Mean (SD)	115 (23.9)
FEV_1_-L, Mean (SD)	2.13 (0.8)
FEV_1_-%, Mean (SD)	83.1 (23.7)
FEV_1_/FVC, Median (IQR)	61.5 (19)
**Additional Pulmonary Functional Test**	
6MWD-meters, Median (IQR)	515 (101)
**Bone-Remodeling Marker Levels**	
CTX ng/mL, Median (IQR)	0.27 (0.21)
Osteocalcin ng/mL, Median (IQR)	14.72 (8.8)
P1NP ng/mL, Median (IQR)	37.6 (20.5)
25-Hydroxy Vitamin D ng/mL, Median (IQR)	20.77 (18.6)
**Densitometry Results**	
Densitometry results according WHO	
Normal, No (%)	37 (25.5)
Osteopenia, No (%)	74 (51.0)
Osteoporosis, No (%)	34 (23.4)
Trabecular bone score, Median (IQR)	1.334 (0.14)
Trabecular bone score <1.300 n, %	55 (38.2)

SD = standard deviation; BMI = body mass index; IQR = interquartil range (25–75 percentile); COPD = Chronic Obstructive Pulmonary Disease; GOLD = Global Initiative for Chronic Obstructive Lung Disease; BODE = Body-mass index, airflow Obstruction, Dyspnea, and Exercise; NETT = national emphysema treatment trial; 6WMT = six minutes walking test; FRAX = Fracture Risk Assessment Tool score; CTX = C-terminal telopeptide of type I collagen; P1NP = N-terminal propeptide of type 1 procollagen; BMD = bone mineral density; WHO = World Health Organization; FVC = forced vital capacity; FEV_1_ = forced expiratory volume-one second; TBS = trabecular bone score; No = number.

Emphysema was visually detected in 56.5% of the population, with centrilobular and paraseptal (47.5%) being the most frequent type, followed by centrilobular alone (44%) and paraseptal alone (8.5%). The majority of the population had low bone mass according to WHO criteria (51%) and 23.4% had osteoporosis. In 13 patients, the BTM levels could not be measured.

Median TBS in our population was 1.334. The distribution of the different categories of TBS levels by the results of DXA scan is shown in Table 2. Importantly, 55 out of 145 (38%) smokers had abnormal TBS values (<1.300) and we also highlight that 8.3% of the patients with normal densitometry values have TBS values in the range of partially deteriorated and degraded microarchitecture. Interestingly, we found that 56 patients (62%) with normal TBS values were diagnosed with osteopenia and osteoporosis using the DXA scan.

**Table 2:** Distribution of the different TBS categories by DXA results.

		Densitometry results according to WHO criteria		
		Normal	Osteopenia	Osteoporosis
**Diagnosis by TBS in column**	**≥1.300**	34 (91.2%)	47 (63.5%)	9 (26.5%)
	**1.200–1.300**	1 (2.7%)	23 (31.1%)	16 (47%)
	**≤1.200**	2 (5.4%)	4 (5.4%)	9 (26.5%)

TBS = trabecular bone score; DXA = dual energy absorptiometry.

Table 3 shows the comparison of smokers with and without COPD. COPD patients were significantly older and had a lower BMI. Moreover, there was a higher proportion of active smokers with a higher smoking history. As expected, emphysema was more frequent and more severe in COPD patients. A main finding was that COPD patients had lower TBS (1.3± 0.13 vs 1.4 ±0.16, *p* = 0.003) compared with smokers without COPD. Comparing BTMs, we only found statistically significant differences in P1NP values (35.2±19.6 vs 41.5±30.6 respectively, *p* = 0.0045).

**Table 3:** Baseline clinical, bone remodeling markers and densitometry characteristics of patients with and without COPD.

Characteristics	COPD (n = 99)	No COPD (n = 46)	p Value
**Demographic**			
Age years-old, Mean (SD)	64.3 (8.1)	60.1 (7.01)	0.003
Male, N_o_ (%)	55 (56.1)	26 (56.5)	0.960
BMI Kg/m^2^, Mean (SD)	26.7 (4.5)	28.2 (4.8)	0.080
**Clinical**			
Active smoker, N_o_ (%)	37 (37.4)	32 (69.6)	0.000
Pack-years of smoking, Median (IQR)	49.3 (31.2)	38.9 (19.7)	0.040
Emphysema, N_o_ (%)	64 (68.3)	18 (39.1)	0.004
Severity of emphysema, by NETT (0–4), Median (IQR)	1 (2)	0 (1)	0.000
Inhaled steroids, No (%)	35 (36)	2 (4.4)	0.000
**Bone-Remodeling Marker Levels**			
CTX ng/mL, Median (IQR)	0.26 (0.19)	0.31 (0.22)	0.141
Osteocalcin ng/mL, Median (IQR)	14.2 (7.9)	16.5 (10.4)	0.221
P1NP ng/mL, Median (IQR)	35.2 (19.6)	41.5 (30.6)	0.004
25-Hydroxy Vitamin D ng/mL, Median (IQR)	20.3 (17.3)	24 (20.4)	0.461
**Densitometry Results**			
Densitometry results according to WHO			
Normal, N_o_ (%)	22 (22.2)	15 (32.6)	
Osteopenia, N_o_ (%)	51 (51.5)	23 (50)	
Osteoporosis, N_o_ (%)	26 (26.2)	8 (17.4)	0.300^a^
Trabecular bone score, Median (IQR)	1.323 (0.13)	1.480 (0.16)	0.003
Trabecular bone score <1.300 n, %	45 (45.9)	10 (21.7)	0.005

COPD = Chronic Obstructive Pulmonary Disease; SD = standard deviation; BMI = body mass index; IQR = interquartil range (25–75 percentile); NETT = national emphysema treatment trial; CTX = C-terminal telopeptide of type I collagen; P1NP = N-terminal propeptide of type 1 procollagen; BMD = bone mineral density; WHO = World Health Organization; TBS = trabecular bone score.

^a^chi2 Test between Osteoporosis diagnosis and study groups (COPD vs no COPD).

Table 4 shows the independent association of each studied parameter with TBS. Age, FEV_1_, higher BMI and number of exacerbations were associated with higher TBS values. Table 5 shows this association but only in those with COPD. The same predictors were found in both groups.

**Table 4:** Linear regression analysis with TBS as a dependent variable in all patients.

Variables	β	IC 95%	P value
**Univariate analysis**			
Age years-old	-0.004	-0.006-(-)0.001	0.003
Male	0.070	0.032–0.107	0.000
BMI Kg/m^2^	0.008	0.004–0.012	0.000
Pack-years	-0.000	-0.001–0.000	0.084
Smoking status	0.021	-0.017–0.059	0.275
Emphysema	-0.060	-0.098-(-)0.021	0.002
Severity of emphysema, by NETT (0–4)	-0.032	-0.049-(-)0.015	0.000
COPD	-0.060	-0.099-(-)0.020	0.004
BODE	-0.029	-0.041-(-)0.017	0.000
Post-bronchodilator FEV_1_ (%)	0.001	0.000–0.002	0.002
DLCO (ml/min/mmHg)	0.002	0.001–0.003	0.000
6MWT meters	0.000	0.000–0.001	0.000
Number of exacerbations	-0.034	-0.050-(-)0.017	0.000
Inhaled CT	-0.060	-0.102-(-)0.178	0.006
Oral CT	-0.135	-0.210-(-)0.060	0.000
CTX-I ng/mL	-0.151	-0.258-(-)0.044	0.006
P1NP	-0.000	-0.001–0.000	0.430
Lumbar spine DMO	0.327	0.247–0.406	0.000
**Multivariate analysis**^a^			
Age	-0.003	-0.007-(-)0.000	0.008
BMI Kg/m^2^	0.005	0.000–0.011	0.032
Male	0.012	-0.044–0.076	0.956
Oral CT	0.022	-0.049–0.093	0.535
Inhaled CT	0.006	-0.036–0.048	0.768
6MWD meters	-0.000	-0.000–0.000	0.830
Post-bronchodilator FEV_1_ (%)	0.001	0.000–0.002	0.012
Number of exacerbations	-0.019	-0.034(-)-0.004	0.015
Emphysema	0.000	-0.043–0.045	0.964
CTX-I ng/mL	0.023	-0.095–0.142	0.695
Lumbar spine DMO	0.332	0.205–0.459	0.000

COPD = Chronic Obstructive Pulmonary Disease; BMI = body mass index; NETT = national emphysema treatment trial; FEV_1_ = forced expiratory volume-one second; DLCO = diffusing capacity for carbon monoxide; 6WMT = six minutes walking test; CTX = C-terminal telopeptide of type I collagen; CT = corticosteroids.

^a^ All the variables included in the multivariable analysis are shown in the table.

**Table 5:** Linear regression analysis with TBS as a dependent variable in COPD patients.

Variables	β	IC 95%	P value
**Univariate analysis**			
Age years-old	-0.003	-0.006–0.000	0.047
Male	0.067	0.024–0.110	0.003
BMI Kg/m^2^	0.006	0.001–0.011	0.022
Pack-years	-0.000	-0.001–0.000	0.440
Smoking status	0.021	-0.024–0.068	0.355
Post-bronchodilator FEV_1_ (%)	0.053	0.026–0.081	0.000
DLCO (ml/min/mmHg)	0.002	0.001–0.004	0.001
BODE	-0.029	-0.041(-)-0.017	0.000
Emphysema	-0.050	-0.097-(-)0.004	0.032
Severity of emphysema, by NETT (0–4)	-0.027	-0.453-(-)0.007	0.006
6MWD-meters	0.000	0.000–0.001	0.000
Number of exacerbations	-0.029	-0.046-(-)0.112	0.001
Inhaled CT	-0.060	-0.1059-(-)0.016	0.009
Oral CT	-0.120	-0.193–0.047	0.002
CTX ng/mL	-0.166	-0.291-(-)0.040	0.010
P1NP	-0.000	-0.002–0.001	0.584
Lumbar spine BMD	0.330	0.241–0.419	0.000
**Multivariate analysis**^**a**^			
Age	-0.004	-0.006(-)-0.001	0.007
Male	-0.007	-0.055–0.039	0.752
BMI Kg/m^2^	0.005	0.000–0.009	0.026
6MWD-meters	-0.000	-0.000–0.000	0.878
Post-bronchodilator FEV_1_ (%)	0.001	0.000–0.002	0.007
Emphysema	0.011	-0.034–0.057	0.625
Lumbar spine BMD	0.319	0.185–0.452	0.000
Number of exacerbations	-0.021	-0.037(-)-0.005	0.011
Inhaled CT	0.004	-0.040–0.048	0.854
Oral CT	0.023	-0.049–0.096	0.519
CTX ng/mL	0.009	-0.114–0.133	0.875

COPD = Chronic Obstructive Pulmonary Disease; BMI = body mass index; NETT = national emphysema treatment trial; FEV_1_ = forced expiratory volume-one second; DLCO = diffusing capacity for carbon monoxide; 6WMT = six minutes walking test; CTX-I = C-terminal telopeptide of type I collagen.

^a^ All the variables included in the multivariable analysis are shown in the table.

## Discussion

The most important and novel information of the present study is that it reports for the first time TBS values in a high risk population of active and former smokers with and without COPD. We found lower TBS in COPD patients and, interestingly, that at least 40% of the studied individuals have abnormal (<1.300) TBS values, which the literature reported to be associated with an increased risk of major osteoporotic fracture, independent of BMD values [19,20,21,22,23]. We also found that the best predictors of TBS values were lung function, number of exacerbations, age and BMI. Moreover, TBS values were, as expected, lower in women than in men, but only in smokers without COPD, after adjusting for other relevant factors.

It is well known that one of the most common extra pulmonary manifestations of COPD is osteoporosis [24,25,26]. The present study showed that patients with COPD attending a pulmonary clinic have lower TBS compared with those without. This is in line with a study performed on 61 COPD males, where BMD and TBS were independently associated with 2 or 3 vertebral fractures in these patients [27]. Similarly, in a study that explores the clinical factors associated with trabecular bone score in 29.047 postmenopausal women from Manitoba, COPD was statistically significant in the multiple linear regression analysis [28]. This is also in agreement with previous findings indicating that COPD patients have reduced bone stiffness and failure load estimated by finite elements analysis based on in-vivo high resolution images of the distal radius and tibia [14]. Furthermore, a recent study in postmenopausal women with COPD demonstrated that the microarchitectural deterioration could be evidenced by low trabecular number, connectivity, and thickness, additionally with an increase in trabecular spacing [29]. Therefore, the evaluation reporting reduced TBS values in these COPD patients could imply that they have decreased bone strength [30], placing them at an increased fracture risk [31,32].

The present work also found that lung function, age and BMI were the most important factors associated with TBS values in this population. Lung function represented by FEV_1_% was one of the most important factors independently associated with TBS values in the entire population and in COPD patients. Previous studies have also demonstrated an association between pulmonary function and bone density or bone architecture in COPD patients [33]. Moreover, Kulak *et al*. showed that patients with a more severe degree of airway limitation (GOLD spirometric grades 3 and 4) presented a significantly lower bone formation rate when compared with patients with mild to moderate disease (0.028±0.009 versus 0.016±0.011 μm^3^/μm^2^/day, p = .04)(29). Misof *et al*., also found that heterogeneity of cancellous bone mineralization was significantly lower in the most severe COPD patients (GOLD III and IV) [34].

As also reported in the general population, lower BMI is associated with a lower TBS [35]. A low BMI is usually present in approximately 15–20% of COPD patients [36] and it also plays an important role in osteoporosis and risk fracture [37,38]. COPD patients are predisposed to low body weight due to multiple reasons including malnutrition, depression and hyper-catabolism related to increased energy cost (breathing, tissue hypoxia, use of beta-agonist, and chronic systemic inflammation) [39,40,41]; therefore, it is not a surprise that the present study results indicate that it is also associated with low TBS.

The independent association between exacerbations and TBS values is another finding from the present study. Exacerbations play an important role in COPD patients because they have negative impacts in several aspects, such as pulmonary function [42,43], health status [10], survival [44,45], the BODE index [46] and also socioeconomic cost [47]. This impact also affects and causes deterioration in risk facts related to osteoporosis. In this regard, exacerbations are associated with physical inactivity [46], systemic inflammation [48] and the use of corticosteroids. A longitudinal study of 42 COPD patients demonstrated that patients with a history of exacerbations have a higher decrease in bone mineral density assessed on chest computed tomography compared with those without a history of exacerbations [49].

Interestingly, TBS is abnormal (<1.300) in a significant proportion of the smokers (40%) which did not exactly match with the ones that had abnormal BMD values (*Table 1*), suggesting the complementary role of this technique in evaluating risk fracture in this high risk population. We found two scenarios that confirmed the complementary information provided by BMD and TBS: 47 (63.5%) patients diagnosed with osteopenia by DXA had normal TBS and 9 (26.5%) osteoporotic patients by DXA scan had normal TBS. These results suggest that these techniques provide different information about bone status, indicating that they are complementary and maybe useful to identify individuals at risk of bone fracture. Further studies should explore the potential complementary role of these techniques to better identify those smokers at high risk for bone fractures.

The present study has limitations. Firstly, this is a highly selected population of smokers from a tertiary academic Pulmonary Clinic; therefore, results should be confirmed in other populations. Secondly, this is a relatively small population lacking a never-smoker control group to complete the entire spectrum of population types, this could have helped us determine the normal values representative of our local population. Thirdly, fortunately these patients are currently under follow up to determine if those with abnormal TBS values and normal bone mineral density will develop bone fractures, enabling us to provide that relevant information.

## Conclusions

A significant proportion of active and former smokers with and without COPD have an affected TBS, an easy way to measure skeletal microarchitecture and therefore, a potential predictor of risk fracture. Lung function, age, BMI and number of exacerbations were their main predictors. TBS seems to provide complementary information in the evaluation of risk fracture in this high-risk population. Further studies should validate these important findings.

## Supporting information

S1 File Clinical questionnaire. Clinical questionnaire in Spanish.
(DOCX)

S2 File Clinical questionnaire in English. Translation of the clinical questionnaire.
(DOCX)
