# Supplementary material for: Trabecular bone score in active or former smokers with and without COPD
Source: PLoS One. 2019 Feb 1;14(2):e0209777. doi: 10.1371/journal.pone.0209777 (PMC6358061; doi:10.1371/journal.pone.0209777)
Supplement: S1 File — Clinical questionnaire in Spanish. (DOCX) [file pone.0209777.s001.docx]

**CUESTIONARIO CLÍNICO**

Número de historia

Fecha visita (día/mes/año)

Fecha nacimiento (día/mes/año)

Peso (Kg)

Talla (cm)

Raza: caucásica/asiática/negra

**Vida fértil**

Edad menarquia (año)

Amenorreas prolongadas premenopausia (≥12 meses): si/no

Nº embarazos

Nº embarazos a término

Nº lactantes

Tiempo total de lactancia (meses)

Anticonceptivos (si/no)

Nº de años de toma de anticonceptivos

**Fracturas previas a la menopausia**

Tipo fractura (Cadera, vertebral...)

Fecha fractura (día/mes/año)

Fue la fractura consecuencia de un traumatismo severo (caída superior a un cuerpo y medio del paciente): si/no

**Fracturas posteriores a la menopausia: si/no**

Tipo fractura (Cadera, vertebral...)

Fecha fractura (día/mes/año)

Fue la fractura consecuencia de un traumatismo severo (caída superior a un cuerpo y medio del paciente): si/no

**Antecedentes familiares (primer grado: padre, madre o hermano)**

Osteoporosis diagnosticada: si/no

Fractura cadera: si/no

Fracturas vertebrales: si/no

Otras fracturas óseas: si/no

Indicar que fractura

**Antecedentes familiares (otros familiares: primo, tío...)**

Tipo de fractura

**Tóxicos**

Tabaco: si/no y desde cuando

Exfumador: desde cuando

Nª cigarrillos día

Alcohol si/no; exbebedor

Cantidad de alcohol diaria (nº dosis al día)

Café si/no; nº tazas al día

**Actividad física/ocio**

Sedentaria (casi todo el día inactivo)

Ligera (algún ejercicio suave 4 veces por semana)

Moderada (algún ejercicio vigoroso 2-3 veces por semana)

Alta (algún ejercicio vigoroso ≥4 veces por semana)

**Actividad física laboral**

Sedentaria (casi todo el día inactivo)

Ligera (algún ejercicio suave 4 veces por semana)

Moderada (algún ejercicio vigoroso 2-3 veces por semana)

Alta (algún ejercicio vigoroso ≥4 veces por semana)

**Exposición al sol**

Muy baja (no sale)

Suficiente (al menos 15 minutos dos veces por semana, brazos y piernas)

Alta (igual o mas de una hora diaria)

**Enfermedades previas**

Endocrinas

Diabetes mellitus 1

Diabetes mellitus 2

Enfermedad de Addison

Síndrome de cushing

Acromegalia

Hiperparatiroidismo primario

Hipertiroidismo

Hipogonadismo (primario y secundario)

Síndrome de ovario poliquístico

Reumatológicas

Artritis reumatoide

Nutricionales

Anorexia/obesidad

Digestivas

Gastrectomía

Celiaquía

Malabsorción

Enfermedad de Crohn

Colitis ulcerosa

Resección intestinal

Inflamatorias

Amiloidosis

Espondilitis anquilosante

Artritis reumatoide

Colagenosis

Hepáticas

Hepatopatía severa

Porfiria congénita

Hemocromatosis

Cirrosis biliar primaria

Neoplasias

Tipo de cáncer

EPOC

Otras enfermedades: indicar nombre

**Tratamiento antiosteoporótico: si/no**

Nombre de medicamento

Frecuencia tratamiento

Duración del tratamiento (año inicio del tratamiento)

Adherencia al tratamiento si/no

**Tratamiento hormonal sustitutivo si/no**

Nombre de medicamento

Frecuencia tratamiento

Duración del tratamiento (año inicio del tratamiento)

Adherencia al tratamiento si/no

**Rehabilitación si/no**

Tipo (calor, fisioterapia, hidroterapia, estimulación eléctrica, corsés…)

Frecuencia (tiempo y días/semanas)

Duración de la rehabilitación, año de inicio de la rehabilitación

**Tratamiento habitual (frecuencia y duración, año inicio del tratamiento)**

Glucocorticoides

Sales de litio

Antiepilépticos

Heparina

AINEs

Tiazidas

Citostáticos y otros (indicar nombre)

**Calidad de vida**

Buena/regular/mala

**Movilidad articulaciones**

Buena/regular/mala

**Pérdida de mas de 3 centímetros de altura (si/no)**
