# Supplementary material for: Trabecular bone score in active or former smokers with and without COPD
Source: PLoS One. 2019 Feb 1;14(2):e0209777. doi: 10.1371/journal.pone.0209777 (PMC6358061; doi:10.1371/journal.pone.0209777)
Supplement: S2 File — Translation of the clinical questionnaire. (DOCX) [file pone.0209777.s002.docx]

**CLINICAL QUESTIONNAIRE**

History number

Date of visit (day/month/year)

Date of birth (day/month/year)

Weight (Kg)

Height (cm)

Race: caucasian/asian/black

**Fertile life**

Menarche age (year)

Prolonged amenorrhea premenopause (≥12 months): yes / no

No. of pregnancies

No. of term pregnancies

Nº of infants

Total breastfeeding time (months)

Contraceptives (yes / no)

No. of years of contraceptive use

**Fractures prior to menopause**

Fracture type (Hip, vertebral ...)

Date fracture (day / month / year)

It was the fracture resulting from a severe trauma (fall greater than a body and a half of the patient): yes / no

**Fractures after menopause: yes / no**

Fracture type (Hip, vertebral ...)

Date fracture (day / month / year)

It was the fracture resulting from a severe trauma (fall greater than a body and a half of the patient): yes / no

**Family history (first degree: father, mother or brother)**

Osteoporosis diagnosed: yes / no

Hip fracture: yes / no

Vertebral fractures: yes / no

Other bone fractures: yes / no

Indicate the type of the fracture

**Other family history (other relatives: cousin, uncle ...)**

Type of fracture

**Toxic**

Tobacco: yes / no, and from when.

Ex-smoker: from when.

Nª cigarettes day

Alcohol yes / no;

Amount of daily alcohol (nº dose per day)

Coffee: yes / no; Nº cups a day

**Physical activity / leisure**

Sedentary (almost all day inactive)

Light (some gentle exercise 4 times a week)

Moderate (some vigorous exercise 2-3 times per week)

High (some strenuous exercise ≥4 times per week)

**Labor physical activity**

Sedentary (almost all day inactive)

Light (some gentle exercise 4 times a week)

Moderate (some vigorous exercise 2-3 times per week)

High (some strenuous exercise ≥4 times per week)

**Sun exposure**

Very low (does not come out)

Enough (at least 15 minutes twice a week, arms and legs)

High (equal or more than one hour daily)

**Previous diseases**

Endocrine

Diabetes mellitus 1

Diabetes mellitus 2

Addison's disease

Cushing's syndrome

Acromegaly

Primary hyperparathyroidism

Hyperthyroidism

Hypogonadism (primary and secondary)

Polycystic ovary syndrome

Rheumatology

Rheumatoid arthritis

Nutritional

Anorexia / obesity

Digestive

Gastrectomy

Celiac

Malabsorption

Crohn's disease

Ulcerative colitis

Bowel resection

Inflammatory

Amyloidosis

Ankylosing spondylitis

Rheumatoid arthritis

Colagenosis

Hepatic

Severe liver disease

Congenital porphyria

Hemochromatosis

Primary biliary cirrhosis

Neoplasms

Type of cancer

COPD

Other diseases: indicate name

**Anti-osteoporotic treatment: yes / no**

Name of medication

Treatment frequency

Duration of treatment (year of treatment initiation)

Adherence to treatment: yes / no

**Substitute hormone treatment yes / no**

Name of medication

Treatment frequency

Duration of treatment (year of treatment initiation)

Adherence to treatment yes / no

**Rehabilitation yes / no**

Type (heat, physiotherapy, hydrotherapy, electrical stimulation, corsets ...)

Frequency (time and days / weeks)

Duration of rehabilitation, year of beginning of rehabilitation

**Regular treatment (frequency and duration, year of treatment initiation)**

Glucocorticoids

Lithium salts

Antiepileptics

Heparin

NSAIDs

Thiazides

Cytostatics and others (indicate name)

**Quality of life**

Good / regular / bad

**Joint mobility**

Good / regular / bad

**Loss of more than 3 centimeters in height (yes / no)**
